# Dark Chocolate: Opportunity for an Alliance between Medical Science and the Food Industry?

**DOI:** 10.3389/fnut.2017.00043

**Published:** 2017-09-26

**Authors:** Ivan M. Petyaev, Yuriy K. Bashmakov

**Affiliations:** ^1^Lycotec Ltd., Cambridge, United Kingdom

**Keywords:** dark chocolate, cocoa flavanols, cardiovascular effects, biotechnology

## Abstract

Dark chocolate (DC) was originally introduced in human nutrition as a medicinal product consumable in a liquid form. Century-long efforts of food industry transformed this hardly appealing product into a valuable modern culinary delight with clear predominance of confectionery brands of DC on the market. However, current epidemiological data as well as multiple experimental and clinical observations reveal that DC consumption may have a profound effect on cardiovascular, central nervous systems, hemostasis, and lipid metabolism. However, despite of growing body of modern scientific evidence revealing medicinal properties of cocoa-based products, DC remains more gourmet culinary item than medicinal food product. Even today there are no clear dietary recommendations on consumption of cocoa flavonoids (flavanols) for health purpose. Clinical trials with DC rarely include monitoring of plasma flavanol concentration in volunteers. Moreover, there is no standardized assay or any quantitative requirements for flavanol content in the commercial brands of DC. High flavanol content is often sacrificed during manufacturing for a better taste of DC due to bitterness of cocoa flavonoids. All these problems including subsequently arising ethical issues need to be addressed by joint efforts of food industry and medical science. Moreover, application of microencapsulation technology in DC manufacturing, as well as molecular selection of best flavanol producers may drastically change bioavailability of DC bioactive ingredients and DC production technology. Nevertheless, only strict causative approach, linking possible health effect of DC to its bioactive ingredients considered as nutraceuticals, may change the current landscape in nutritional research related to cocoa-based products and create a trustworthy path for their medicinal use.

## Introduction

Dark chocolate (DC) was introduced into the human diet in South America at least 3,000 years ago and was brought to Europe by Christopher Columbus (1). The first specimens of the cocoa tree were transported to Spain by the end of the sixteenth century and were classified as *Theobroma cacao* by Carl Linnaeus in 1753 (2–4). In Pre-Columbian cultures, cocoa products were believed to be of divine origin and were consumed exclusively in beverage form as a remedy for fatigue, indigestion, and gastrointestinal disorders (5, 6). European dietary culture developed on Christian traditions was somewhat suspicious and xenophobic toward foreign dietary innovations, opposing the wide introduction into the human diet of coffee, tea, and cocoa beverages during the later Medieval and Renaissance periods (3). Nevertheless, the medicinal properties of cocoa as an expectorant, diuretic, anti-depressive, weight gaining stimulant, and aphrodisiac were proclaimed in the sixteenth and seventeenth centuries (7, 8). As a result, multiple modifications of cocoa bean processing (drying, heating, and pulverization) and preparation (addition of sugar and spices) took place in small pharmacies and food shops in order to improve the bitter taste of cocoa beverages (8). As reported in 1662, British soldiers stationed in Jamaica consumed a solidified cocoa bean paste containing sugar, anise, vanilla, cinnamon, and almonds, marking the birth of DC (3). Altogether, these modifications drastically changed the taste of cocoa-based products transforming them from a medicinal product to the culinary delight.

Despite centuries of research into the health benefits of DC, most of the studies conducted in the past hardly meet the requirements of modern medical science and appear inconclusive. However, stricter guidelines for clinical trials, advances in medical statistics, and the enormous progress in molecular medicine have very recently shaped a solid scientific background to the medicinal use of DC. There are multiple questions and challenges relating to the habitual consumption of DC by individuals with risk of cardiovascular disease (CVD) and CVD patients. Many of these can be successfully addressed and resolved by the joint efforts of modern medical science and the food industry.

## The Darker the Better

Cocoa solids are intermediates of chocolate manufacturing, forming after cocoa butter extraction from the cocoa beans. Cocoa solids, called otherwise cocoa powder, confer a dark color to DC (9). In recent times the food industry produces three types of chocolate: (a) DC, prepared mostly from cocoa bean solids (up to 80% of total weight) with the addition of cocoa butter; (b) milk chocolate, derived from high-fat milk with additions of sugar and low amounts of cocoa bean solids (<10% of total weight); and (c) white chocolate, based on cocoa butter, milk, and sugar with no cocoa solids (9, 10). Most of the health benefits are attributable to the consumption of DC, while milk and white chocolate reportedly have no considerable beneficial impact on health (9–11). The overwhelming majority of food science reports have been focused on improvement of taste, texture, appearance, and shelf life of cocoa-based products, whereas the physico-chemical characteristics of chocolate predetermining its health benefits remained largely unknown until the end of last century. However, emerging pieces of evidence related to the health benefits of cocoa products, in particular the effects of DC on cardiovascular health, paved the way for studies focused on identifying the DC bioactive compounds.

**Figure 1:**
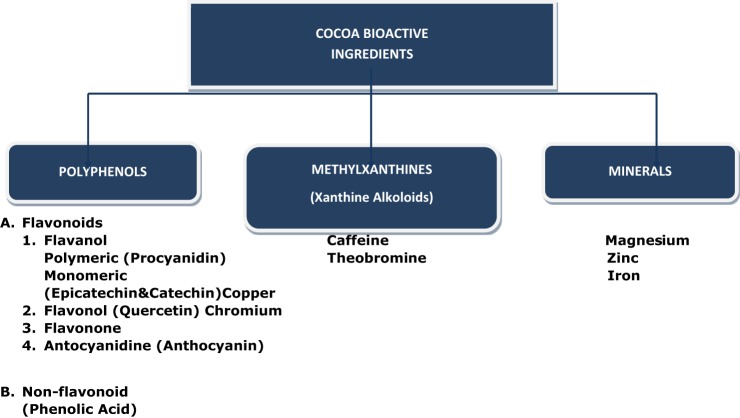
Cocoa bioactive ingredients.

Cocoa beans contain more than 300 identifiable chemical compounds (12). Many other substances appear in the DC matrix during fermentation, roasting, and processing of cocoa beans (13, 14). Scrupulous identification of the biologically active ingredients over the last decade has revealed that there are at least three groups of substances in cocoa beans with potential health effects. These are flavonoids (epicatechins and procyanids), theobromine/caffeine, and minerals—magnesium, iron, and zinc (Figure 1). Some other as yet unidentified compounds may contribute to the health benefits of DC (9, 15).

Although theobromine, caffeine, and minerals have distinct and independent effects on the cardiovascular system as discussed elsewhere, there is a general consensus in modern nutritional science that flavonoids (13, 15, 16) are the major group of bioactive compounds mediating the effects of DC in CVD. Since flavonoids are found predominantly in cocoa solids, cocoa-enriched DC is widely assumed to have a higher bioefficacy due to higher flavonoid content and, therefore, higher antioxidant activity (13, 15, 16). As a result, most of the current interventional studies are performed using DC containing up to 80% cocoa solids.

## Cocoa Flavonoids: Their Action and Bioavailabilty

Cocoa flavonoids belong to a large class of dietary polyphenols present in fruits and vegetables. Flavonoids comprise about 12–18% by dry weight of the cocoa beans (17). Cocoa flavonoids confer a bitter taste to cocoa beans, making them revolting to human taste in unprocessed form. However, during manufacturing, the amount of polyphenols in cocoa beans may become significantly reduced thereby affecting the antioxidant properties of the final cocoa products (18). In their chemical nature, cocoa flavonoids are flavan-3-ols, this is why they are often referred to as flavanols. Flavanols are further categorized depending on their structure as catechin, epicatechin (monomers), and proanthocyanidin oligomers (19, 20). Proanthocyanidins make up >50% of the total flavonoid content in cocoa beans while catechins and anthocyanins comprise about 37 and 4%, respectively (20).

**Figure 2:**
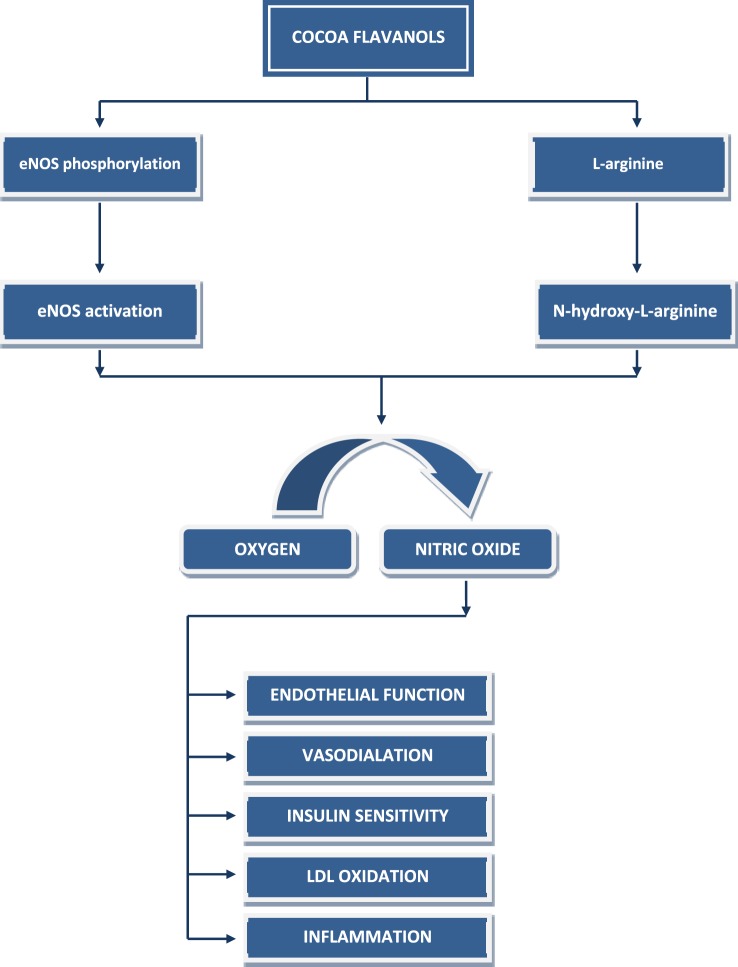
Cocoa flavanols and nitric oxide pathway.

The molecular mechanism (Figure 2) behind the action of cocoa flavanols is primarily connected to their effect on the nitric oxide-mediated pathway, resulting in nitric oxide production *via* Ca(2^+^)-independent eNOS activation/phosphorylation (21). Moreover, cocoa flavanols decrease degradation of nitric oxide and increase availability of l-arginin as a NO donor (21, 22). This universal mechanism is believed to cover most of the physiological effects of chocolate flavanols on the cardiovascular and nervous system (23).

In general, bioavailability of dietary flavonoids is fairly limited due to their short half-life, hydrophobicity, and susceptibility to oxidation (24). The absorption rate of cocoa flavanols is greatly influenced by interaction with the food matrix and co-ingested constituents (24, 25). Theobromine and epicatechins can be efficiently absorbed in the small intestine, whereas proanthocyanidins and oligomeric procyanidins are known to have a limited rate of intestinal absorption and become absorbable only in the colon after metabolic transformations by intestinal microbiota (24–26). Absorption rate of cocoa polyphenols is highly dependent on polymerization rate and unabsorbed portion of polyphenols undergo fecal elimination (26). A lipid environment promotes bioavailability of cocoa flavanols in the intestinal lumen due to possible micellization of polyphenols (27). Epicatechins can be detected in the plasma of volunteers 2 h after DC consumption and have a relatively high clearance rate comprising 2–4 h (28). Therefore, postprandial assessment of bioaccessible fraction of polyphenols in blood seems to be more valuable than information revealing total phenolic content in cocoa-based products.

## DC and Caridovascular Health

The first considerable epidemiological evidence suggesting a possible relationship between DC consumption and cardiovascular health came in 1992 from the Dutch Zutphen Elderly Study published in The Lancet. The study reported an inverse association between dietary flavonoid consumption, hypertension, and cardiovascular death rates in a large cohort of elderly volunteers (29). Extremely interesting information has been obtained from mortality pattern analysis in the Kuna Indians of Panama. Widespread consumption of cocoa flavanols consumed as a beverage in the Kuna Indians provides an intake of up to 900 mg/day of cocoa flavanols and coexists with a significantly reduced rate of CVD and diabetes-related mortality (30). Lower values for the systemic blood pressure over the lifetime of the Kuna Indians were reported by others (31) and providing another piece of evidence supporting epidemiological evidence for the Panama study. The subset of epidemiological research was strengthened by the IDEFICS study which reported that consumption of DC in childhood appears to affect clustered CVD risk factors in European populations (32). The inverse association between DC consumption and coronary heart disease was also reported in the general population of the USA (29, 30). However, some other results suggest that the inverse association may be much stronger for stroke than myocardial infarction (33, 34).

Interventional studies provide further evidence supporting the link between cardiovascular health and DC consumption. Recently performed meta-analysis (42 acute or short-term studies, 1,297 participants) indicates that regardless of the daily amount, regular consumption of cocoa-based products significantly improves flow-mediated dilation (FMD) and reduces systemic blood pressure (35). Although the blood pressure reducing effect of DC seems to be modest and rarely exceeds 3–4 mmHg, the degree of statistical significance and magnitude of changes in blood pressure reflect the amount of cocoa flavanol ingested and the duration of intervention (36, 37). However, the seemingly small impact of cocoa flavonoids on blood pressure could be translated into enormous public health benefits. In the best case scenario assessed by the Markov model (38), regular consumption of DC by individuals with metabolic syndrome may have a significant impact of cardiovascular health, reducing probability of cardiovascular events by 85 per 10,000 population. Despite some legitimate concerns regarding best case scenario epidemiological modeling, these results encourage further research.

Moreover, there is a growing number of smaller interventional studies suggesting that DC consumption may improve endothelial function, decrease arterial stiffness index and aortic pulse wave velocity, reduce platelet adhesion, and improve inflammatory parameters and brachial artery FMD in CVD patients (39–43). It is very encouraging that most of the clinical observations can be reproduced to some extent in experimental settings (10, 12, 26).

## Effect on Neurological and Cognitive Functions

In general, it is believed that dietary polyphenols may have a measurable and reproducible effect on neurological functions (26). However, the impact of cocoa bioactive compounds on the central nervous system (CNS) in particular remains poorly understood and requires further investigation. The ability of cocoa polyphenols to modulate nitric oxide production may represent a major mechanism explaining effects of DC on CNS. Vasodilation and subsequently increased cerebral blood flow promotes oxygen and glucose delivery to the neurons enhancing thereby their function and blood vessel formation in the hippocampus (44). The possible effects of DC on neurological functions may also originate from the antioxidant properties of cocoa polyphenols. Age-related cognitive deterioration and certain neurodegenerative disorders, including Alzheimer’s and Parkinson’s diseases, are closely related to the accumalation of reactive oxygen species (ROS) in the brain (45, 46). Therefore, the preventive effect of cocoa polyphenols on various molecular events initiated by ROS (inhibition of mitochondrial complex I, activation of caspase-3, and apoptosis) reported in different experiments might be explained by the anti-radical properties of bioactive components in cocoa (44, 46). Supplementation of mice with a diet containing cocoa polyphenols and theobromine has also been shown (47) to enhance cholinergic and catecholaminergic transmissions in brain and cause an increase in superoxide-dismutase activity, reversing thereby the metabolic changes associated with neurodegeneration. In mouse hippocampal sections, cocoa extracts have also been shown to reduce oligomerization of amyloid peptide β which is a keystone feature of Alzheimer’s disease (48).

The effect of cocoa bioactives on signaling pathways in neurocytes may provide another rationale for linking DC to regulation of brain functions. Cocoa flavanols and methylxanthines have been shown to affect the activation cascade in phosphatidylinositide 3-kinase/protein kinase B and target of rapamycin signaling pathways (49–51). These play a crucial role in synaptic function, neuronal growth, mechanisms of memory as well as in pathogenesis of neurodegenerative disorders (52). Anti-inflammatory action of cocoa bioactives is another feature which may contribute to the neuroprotective effect of DC (53). As reported, acute ingestion of DC may decrease concentration of adhesion molecules and 4-series leukotrienes in serum, nuclear factor κB activation in leukocytes and the expression of CD62P and CD11b on monocytes and neutrophils in volunteers (54). However, the reproducibility and magnitude of these changes needs to be further investigated. Other experimental studies suggest that cocoa flavanols may reduce cytokine production (55, 56). Suppressed cytokine production caused by DC/cocoa flavanol intake has been shown to be accompanied by inhibition of indoleamine 2,3-dioxygenase, an enzyme controlling tryptophan degradation (56). Therefore, increased availability of tryptophan for serotonin synthesis in the brain after DC intake may result in the enhancement of serotoninergic stimulation in neurons associated with improved mood and cognition (56). This pathway may establish a new molecular paradigm connecting DC consumption, mood, and cognitive function.

Clinical results relating to the effect of DC on mood and cognitive function are rather controversial. There is a certain degree of reproducibility in reports describing increased cerebral blood flow including brain areas responsible for cognition following cocoa flavanols intake (57, 58). However, it is still debatable if acute or chronic DC/cocoa flavanol intake has a measurable impact on mood and cognitive performance (59–61). The discrepancies in these results may reflect variability in the pre-existing health status of volunteers, different spectrum of flavanols used, as well as differences in the cognition assessment protocols.

## Caloric Burden Versus Health Benefits

High sugar content and excessive caloric value of cocoa-based products lead negative perception of chocolate by dieticians (62). Indeed, some trials include ingestion up to 100 g of DC daily thereby providing up to 50 g of carbohydrates, 35 g of fat, and 600 cal (63). However, it was shown recently by the HELENA study that higher chocolate consumption is associated with lower fat deposition in European adolescents (64). In addition, cocoa polyphenols are shown to reduce biosynthesis and intestinal absorption of lipids and carbohydrates (65). Restoration of insulin sensitivity and the hypoglycemic action of DC has been reported by others (22, 66). Thus, the perception of chocolate as a nutritional factor promoting obesity is not justified at least in the case of DC.

However, high sugar content does not have to be an indispensable feature of DC. A similar perception of DC taste and sweetness can be achieved by replacing sugar with inulin, a prebiotic known for its ability to increase mineral absorption and reduce intestinal infection and colon cancer rates (67). Moreover, inulin controls the diversity of the intestinal microbiota by increasing representation of bifidobacteria (68). Therefore, an inulin-containing DC could become a valuable prebiotic product with a potential use in the management of CVD. Such an assumption becomes plausible in the wake of recent discoveries revealing the keystone role of the intestinal microbiota in CVD (69, 70).

## Hepatic Bioavailability as a New Mode of Cocoa Flavanol Action

Ingested polyphenols are known to be widely distributed among internal organs and tissues (digestive system, endothelial cells, heart, kidney, skin, and others) (71). Chocolate flavanols may directly influence some hepatic functions. In particular, catechins and proanthocyanidins are shown to affect lipid turnover in liver *via* SREBP pathway (72). Chocolate polyphenols have also distinct activity on insulin signaling and hepatic glucose production (73). Moreover, they increase hepatic ApoAI transcription and reduce oxidative stress often in dose-dependent manner in cultured hepatocytes (74, 75). These results are consistent with clinical observations revealing hypolipidemic action of cocoa polyphenols and their effect on glucose homeostasis (76). Therefore, the hepatic mode in cocoa polyphenol action becomes an emerging reality although is poorly understood and remains to be thoroughly investigated in future studies.

Several liver-targeted delivery systems have been recently developed (77). In particular, the lycosome hepatic delivery technology (78) employs a network of carotenoid receptors highly expressed on hepatocytes for targeted delivery of bioactive compounds to the liver. This microencapsulation protocol has been recently successfully applied for hepatic delivery of stilbene polyphenols (resveratrol), some hydrophobic peptides from whey protein, and simvastatin, an inhibitor of HMG-CoA reductase (79–81). Lycosome technology was also applied for DC production. As we reported (82), lycosome-formulated DC has a superior ability in the reduction of blood pressure and plasma lipids when compared with regular formulation of DC with similar cocoa flavanol content.

## Conclusion: Transforming DC into Nutraceutical Product

Recently, DC has been credited with a health status in Europe (83). However, this declaration may be premature and imposes substantial obligations on both food industry and medical science to become a reality. As mentioned in the Lancet Editorial (84) almost decade ago and remains true today chocolate represents more food than medicine. Indeed, huge variability in flavanol content and cocoa processing, absence of clinically justified recommendations on cocoa flavanol/DC, as well as obvious predominance on the market of confectionary DC brands with unknown polyphenol content undermine nearest perspectives for medicinal use of DC. Currently, DC consumption remains astonishingly low even in the European countries, where flavonoid intake arising from chocolate consumption accounts for about 1/600 of daily flavonoid intake from all other sources (85).

Transformation of DC into nutraceutical product brings multiple ethical and financial challenges for manufacturers and medical science. It needs to start with agricultural practices and selection cultivars. Selection of *T. cacao* genomic variants with best flavor profile is under way (86). This approach becomes conceivable after recent genome-based categorization of *T. cacao* cultivates into 10 major clusters (87, 88). The genomic-based search for best flavanol spectrum producers using molecular biology holds enormous promise for medicinal use of DC. However, selection of cultivars suitable for medicinal use requires a precise knowledge of flavanol spectrum conferring bioefficacy for DC. Unfortunately, this information is hardly available now since most of the clinical trials do not provide scrupulous information revealing flavanol content neither in DC products nor plasma of patients before and after treatment.

Moreover, DC production technologies need to be reevaluated from the standpoint of modern food chemistry. Up to 90% of cocoa flavanols are known to be lost in the cocoa beans during post-harvest processing (88). Since taste and flavor improvement remains a major motive for innovations in chocolate manufacturing, reduced flavanol content resulting in diminished bitterness of DC often concurs with interests of chocolate manufacturers, limiting thereby bioefficacy of DC final product. However, taste and flavor should not be sacrificed for high flavanol content. As recently shown, microencapsulation of cocoa flavanols increases their bioavailability and masks sensory perception of flavonoids (25, 89). Modern microencapsulation technologies can be applied for cocoa polyphenols to enhance probiotic properties of DC (90, 91). All these challenges can be successfully addressed by joint efforts of food industry and medical science.

## Author Contributions

Both authors contributed equally into gathering of factual data and writing the manuscript.

## Conflict of Interest Statement

The authors declare that the research was conducted in the absence of any commercial or financial relationships that could be construed as a potential conflict of interest.
